# The Role of Social Context in Shaping Student-Athlete Opinions

**DOI:** 10.1371/journal.pone.0115159

**Published:** 2014-12-18

**Authors:** James N. Druckman, Mauro Gilli, Samara Klar, Joshua Robison

**Affiliations:** 1 Department of Political Science, Northwestern University, Evanston, IL, United States of America; 2 Department of Political Science, University of Arizona, Tucson, AZ, United States of America; 3 Department of Political Science, Aarhus University, Aarhus, Denmark; Leibniz Institute for Prevention Research and Epidemiology (BIPS), Germany

## Abstract

How do student-athletes form opinions? This is a particularly important question given ongoing debates about whether student-athletes should be paid and/or allowed to unionize. These debates concern the rights and benefits accrued directly to student-athletes, and thus, understanding their attitudes is of obvious import. Yet, virtually no recent work has delved into how student-athletes form opinions on these issues. We fill this gap with a theoretical framework that predicts changes in social context alter opinions. This leads to the hypothesis that opinions will change once a student-athlete completes his/her career and finds him/herself in a distinct social network. We test the prediction with a survey, implemented in 2012, of one of the most notable athletic conferences in the National Collegiate Athletic Association (NCAA): the Big Ten. We find that post-career student-athletes demonstrate higher levels of support for pay for play and unionization. Our results suggest that student-athletes' opinions seem to depend on their extant social contexts. While our data, from 2012, neither speak to current opinions – given the quickly evolving landscape of college athletics – nor demonstrate what reforms may be “best,” they do accentuate the power of social context in shaping student-athletes' attitudes.

## Introduction

How do college athletes form opinions? Answering this question is of increasing importance given ongoing debates about whether student-athletes should be paid (i.e., pay for play) and/or allowed to unionize. Indeed, these issues have far from trivial stakes. The National Collegiate Athletic Association (NCAA), for instance, currently accrues over $770 million dollars each year from television contracts [1]. Tickets to Division 1 men's basketball games alone bring in an estimated $82.3 million [2]. In addition, many universities directly receive a substantial amount of revenue from their athletic programs, as illustrated by the $77.9 million garnered by the University of Texas from its football program [3]. Pay for play and unionization would both directly affect student-athletes; moreover, the latter would require student-athletes to vote in favor of a union, whereby they would then be treated as employees rather than students. To the best of our knowledge, only two studies have systematically explored student-athlete opinions concerning these issues [4], [5]. Both studies were completed over a decade ago, when National Collegiate Athletic Association (NCAA) revenues were much smaller than they are now. Furthermore, the samples employed were limited – either by focusing exclusively on basketball players [4] or by using a very small subset of student-athletes [5].

In what follows, we investigate the opinions of contemporary student-athletes on the questions of pay for play and unionization. We broaden extant work by examining *the dynamics* behind attitude expression. We theorize and find, via a 2012 survey, that the social context student-athletes experience upon the completion of their careers appears to cause a shift in attitudes: post-career student-athletes become more supportive of pay for play and unionization. While our data, from 2012, neither speak to current opinions – given the quickly evolving landscape of college athletics – nor demonstrate what reforms may be “best,” they do accentuate the power of social context in shaping student-athletes' attitudes.

### Opinion Formation and the Social Environment

The social environment in which individuals find themselves is a common determinant of the opinions they ultimately adopt [6], [7], [8]. As Sinclair states: “Social relationships define our fundamental human experience, from our sense of self to our preferences… [Individuals] are the products of their social environment. They reflect…norms of their social networks and not their own individual preferences” [9], pages xi, xvi. Similarly, Visser and Mirabile [10], page 780 explain, “people's…attitudes are socially structured, reflecting not only their own thoughts and feelings but the preferences of important others as well.” The social environment can be a particularly powerful influence for younger individuals who have yet to form strong opinions [11], [12]. This has the implication that understanding the opinions of college student-athletes requires recognition of the major forces in their social networks.

In the context of college athletics, a defining force in student-athletes' environments are their teams: the NCAA, coaches, and athletic administrators who not only shape their playing careers but also provide guidance on their academic schedule and day-to-day collegiate experience (for example, meals, practice schedules, day-to-day logistics, academic tutoring, yearly financial scholarships, and so forth). Thus, a starting point is to consider the opinions of NCAA officials, coaches, and others in the athletic department.

When it comes to unionization and pay for play, all signs suggest a social environment constituted ostensibly by powerful individuals who oppose these outcomes, even if they support some reforms such as smaller stipends, unlimited food, and guaranteed scholarships [13]. This supposition is sensible given that universities would lose revenue if these changes were adopted. Furthermore, unionization and/or the direct payment of student-athletes would fundamentally change the nature of a university's relationship with its student-athletes by making them more responsible for the athletes' long-term well-being. This would likely involve costly additional programs such as improved medical coverage and larger stipends. The additional costs, in turn, could alter the very nature of university sports programs as schools may no longer be able to financially support a large number of sports teams.

The only systematic empirical evidence we know of concerning administrator opinions on these issues stems from Schneider's survey of administrators and coaches in a Division I athletic conference: only 28% of these respondents supported direct cash payments to athletes [5]. More recent anecdotal evidence suggests general opposition among the NCAA and universities. For example, NCAA President Mark Emmert stated his view that student-athletes should not be converted into paid employees. To him, “one of the guiding principles (of the NCAA) has been that this is about students who play sports” [14]. Moreover, when it comes to unionization, the NCAA chief legal officer stated that “this union-backed attempt to turn student-athletes into employees undermines the purpose of college – an education” and that “we strongly disagree with the notion that student-athletes are employees…” [13], [15]. Opposition to significant pay for play and/or unionization appears to carry over to most Universities [16], [17], [18], [19], [20], [21], [22]. Even in light of recent reforms that will enable schools from five conferences to offer full cost of attendance scholarships (that cover food, clothing, etc), *inter alia*, opposition to direct payment remains: “The move falls short of giving athletes in the high-revenue sports full salaries… To college leaders, such reform would dredge up the dreaded *E*-word. Athletes would serve as employees, which administrators have determined is incompatible with education” [21], page 1. More succinctly, the Big 10 commissioner asserts, “I do not think pay for pay is right. I do not think unions are the answers” [22]: page 3

To understand how this environment may affect student-athletes' opinions, we turn to a theory known as the “spiral of silence” [23]. Scheufele and Moy summarize the theory: “[i]ndividuals constantly scan their environment for present and future distributions of public opinion ‘in order to see which opinions and modes will win the approval of society and which will lead to their isolation'” [23], [24], page 5. Put another way, individuals turn to their immediate social milieu to gain a frame of reference for forming opinions, and they often adopt the *perceived* views of those from whom they hope to gain approval. Subsequently, the attitudes held by those in their immediate social context, and particularly those of powerful individuals within that setting, exert pressure over opinion formation [24].

While the relevance and accuracy of the spiral of silence is debated, it explains opinion formation well in particular environments where social pressure is evident and individuals do not hold strong prior opinions [25], [26]. This describes the setting in which student-athletes find themselves. As discussed, the central social environment for these individuals is their team. The most influential individuals within this context are likely the NCAA, coaches and others in the administration who shape the day-to-day life of student-athletes. The spiral of silence theory thus suggests that since the central frame of reference for current student-athletes consists of individuals who oppose pay for play and unionization, these players should tend to hold the same opinion, i.e. opposition.

On the other hand, once a student-athlete leaves college or completes his/her playing career, their social environment shifts to include friends (presumably from outside the team environment), family, and co-workers, whereas administrators and coaches play a less focal role. The distribution of opinions in this environment will undoubtedly vary. However, it is reasonable to expect that, on average, the sentiments observed in such an environment will not be as clearly opposed to pay for play and unionization as in the former team environment which could weaken the social norm of being opposed to these policies. Upon entering the post-career social environment, retired student-athletes may further reflect on their experiences. Thus, the spiral of silence theory predicts that support for pay for play and unionization will significantly increase once an individual completes his or her career as a student-athlete. This prediction is consistent with the timing of previous unionization and pay for play efforts. Former Northwestern University quarterback Kain Colter, for example, sought unionization in earnest once his playing days were over, although he had previously expressed support for it. Similarly, former NCAA basketball player Ed O′Bannon sued the NCAA and others for profiting from using his likeness without compensating him long after his college playing days had ended. O′Bannon won the first stage of his suit with a federal judge ruling for limited compensation (but not lifting a ban on commercial endorsements). The NCAA is appealing the decision.

We emphasize the theory is based on the perceptions of the reactions in one's social environment – whether these perceptions are accurate is another question that is normatively important (e.g., do the NCAA, universities and athletic departments create the environments perceived by the student-athletes?). Additionally, nothing in our theory or the data we next present allow us to make statements about what is “best” for student-athletes, universities, or the NCAA; rather, the data allow us to understand the dynamics behind opinion formation.

## Data and Methodology

Our survey focuses on the NCAA Big Ten conference, which is located primarily in the Midwest, with Nebraska as the western-most point and Penn State to the east (circa 2013, which is relevant since the conference expanded in 2014). Despite its name, the Big Ten included, at the time of our survey, twelve major universities, all of whom compete in Division I NCAA Athletics. While we recognize the limitations of restricting our sample to only one conference, the Big Ten conference is a strong starting point as it includes a large amount of variance among universities and includes schools that recruit nationally (for another fruitful study of a single conference, see [27]).

In the spring of 2012, we accessed the athletic websites of all Big Ten schools and obtained the full rosters for all sports at every school. We then accessed each school's website to locate and record the e-mail address of every student-athlete listed on those rosters (thus our sample was contingent on the validity and upkeep of the universities' websites). This information was publicly available at all schools except for the University of Nebraska. We contacted officials at the University of Nebraska to obtain directory information for their student-athletes but were declined, and thus they are excluded from our sample. We conducted the survey *prior* to the well-publicized unionization efforts led by Northwestern University quarterback Kain Colter, and we had *no* knowledge that this would occur (i.e., the affiliation of two of the authors with Northwestern is entirely coincidental).

Overall, we located 6,375 names on the rosters. We were unable to find e-mail addresses for 479 student-athletes; therefore, we sent out 5,896 e-mails. Of those, 1,803 bounced back as no longer in service (which could be due to the students no longer being enrolled, database errors, website errors, data entry errors, or another reason). Thus, we successfully sent a total of 4,093 e-mails that, to our knowledge, reached their intended targets. We also sent out one reminder to all respondents. Sample size varied across schools in part due to variations in the number of sports each school sponsors (e.g., at the time of the survey, Ohio State fielded 37 total teams, Michigan had 27 teams, while Northwestern had just 19 teams). We received 1,303 responses leading to response rate, based on the sampling frame, of 1303/4093 = 31.8%. This rate exceeds the typical response rate in e-mail surveys of this length, especially for those that do not employ incentives [28], [29], [30].

While we found notable variance in the success of our e-mails reaching their targets (i.e., not bouncing back), of the 4,093 e-mails that were ostensibly received, we found near identical response rates (i.e., response rates given the sampling frame) across universities with a minimum response rate of 29.59% at Michigan State, a maximum rate of 35.57% at Wisconsin, and an average response rate of 31.84%. In terms of the sample make-up, the following lists the number of respondents and the percentage of the sample that came from each University: Indiana (128; 9.82%), Ohio State (122; 9.36%), Illinois (104; 7.98%), Minnesota (120; 9.21%), Michigan State (100; 7.67%), Purdue (100; 7.67%), Iowa (110; 8.44%), Wisconsin (154; 11.82%), Northwestern (122; 9.36%), Pennsylvania State (116; 8.90%), and Michigan (127; 9.75%). Our responses came from more than twenty-four different sports (specific breakdown is available from the authors). The highest responding sport was track and field which made up 15.12% of the respondents, followed by swimming with 12.97% of the respondents. All other sports consisted of less than 10% of the respondents. Overall, women responded at a higher rate (40.21% versus 21.83%), which is common for web surveys [28], [30].

While our sample may not be perfectly representative of Big Ten student-athletes, it provides a telling view of the opinions of student-athletes given the diversity of the schools sampled and that we have no reason to suspect they differ in terms of reporting relative to other conferences/sports. The demographics of our sample are as follows: 60.94% female; 6.29 African American; 27.4% in the first year of school, 26.3% in their second year, 23.4% in their third year; 22.3% in the fourth year, and less than 1.0% in years beyond four years; 4% having family incomes below $30,000, 16% between $30,000 and 69,999, 26% between $70,000 and $99,999, 35.5% between $100,000 and $100,999, and 18.5% $200,000 or over; and 51% on athletic scholarship.

The timing of our survey was critical for two reasons. First, as mentioned, it took place well before the widespread discussion of unionization and the relevant legal rulings in winter/spring of 2014; it also far preceded reforms by five major conferences that will expand benefits but do not include direct payments or unionization. Second, it took place late enough in the spring that *anyone who reported being a senior had completed their playing days at the school* (we implemented the survey after all sports had completed their seasons). This enabled us to test our hypothesis that those who have completed their college careers would report distinct views as compared with those currently active in their careers.

In terms of the relevant measures, we asked respondents two key questions. The first focused on pay for play: “Recently, a proposal has been made that student-athletes should be allowed to receive pay beyond their scholarship (e.g., that covers tuition and housing). To what extent do you oppose or support this proposal?” with answers on a fully labeled 7-point scale ranging from strongly oppose to strongly support. The second question concerning unions asked: “To what degree do you support or oppose efforts to unionize college athletics?” with answers on a fully labeled 7-point scale ranging from definitely oppose to definitely support. We also included a variety of other measures including gender, race, family income, partisan identification, political ideology, sport played, scholarship status (i.e., does or did the respondent hold an athletic scholarship?), and most importantly, year in school. This latter variable allows us to test our prediction since, as mentioned, given the timing of our survey, anyone with senior status would have completed their college careers. In what follows, we use the terms “senior” and “post-career” interchangeably.

We obtained Northwestern's Institutional Review Board approval (prior to the study) for the consent procedure to proceed such that the e-mail invitation stated, “Completing the questionnaire will be considered your consent to participate.” Thus, it was a written consent via agreement to participate. We received approval from the Review Board for the consent procedure and the survey.

## Results

Overall, we find that 49.29% of the respondents support pay for play (i.e., they reported a score higher than 4 which meant they supported it at some level, since 4 on both scales indicated “neither oppose nor support” and all scores higher than 4 indicated some level of support) and 22.02% support unionization. That nearly half of the sample supports pay for play suggests higher support relative to the general public; for instance, 33% of respondents in a national survey registered support for the payment of athletes in a *Washington Post-ABC News* poll [31].

The level of support for direct payment of athletes in our sample is substantially lower than found by Schneider [5], who reported 86% support for direct cash payment of athletes. However, Schneider's sample was skewed toward male juniors and seniors, which is a demographic that is particularly likely to be supportive of this policy. Moreover, Schneider only focuses on basketball and football players: athletes who would presumably benefit the most from a payment policy given that they are on teams that generate the most revenue. Our sample includes athletes from a much wider variety of teams and, as a result, our results are more in line with those reported by Sack [4]. He reports that 43% of respondents to an NCAA-sponsored survey agreed or strongly agreed that student-athletes deserved a share of TV revenues, and 40% indicated that college athletes should have the right to the same benefits as other workers. As with Schneider's study, the sample explored by Sack is narrow in that it only focused on male and female basketball players. Our results provide a more comprehensive account of student-athlete opinion on this issue. As we will note below, the relatively low level of support for unionization that we find likely reflects a lack of knowledge of unionization efforts, since our survey took place prior to 2014 unionization efforts. More importantly, we note the relative difference between support among seniors and non-seniors, as we will discuss further.

We directly test our hypothesis by regressing each key dependent variable in an ordered probit model on a dummy variable indicating whether or not the respondent reported being a senior and thus having completed his/her career. Notice the sample size (*n*) shrinks relative to the total response rate due to non-responses on selected variables in the analyses. We also include the aforementioned demographics to control for systemic differences along socio-demographic cleavages, as well as variables indicating membership on the football team or men's basketball team. Given that men's basketball and football teams are major revenue generating sports, it is plausible to conclude that team members in these particular sports may feel more entitled to payment and support. Finally, we include a variable that indicates whether the respondent holds/held an athletic scholarship.

We present results from *unweighted* data, though we also explored the results using weights. Since we have all 6,375 e-mail addresses to which we attempted to send the survey, which include both the name of the university and the name of the potential respondent, we are able to impute gender based on the individuals' first names. We regressed (using a probit model) “response” on gender and a dummy variable for each school. From those results, we used *Clarify* [32] to compute probability of response by school and gender. (These probabilities are based on the entire population, ignoring failed e-mails, and not the sampling frame which was the basis for computing the reported response rate; the overall response rate based on the population is 20.44%.) Following the propensity weighting approach [33], we then took the inverse of these probabilities (using averages for survey responses where gender was not clear) to weight the regressions. In every case, the central substantive and statistical results replicate what we next report.

We report the unweighted results of our regressions in Table 1. We find support for our hypothesis, with the year of college (i.e., senior) having a significant and substantively large impact on support for pay for play and unionization. To assess the substantive impact, we hold all other variables at their mean levels, and compute the probability of supporting pay for play and then unionization (i.e., a score higher than 4, as explained) for non-seniors compared to seniors. The probability of supporting pay for play jumps from 46.74% for non-seniors to 66.56% for seniors. A similarly large jump in observed support occurs on the unionization measure, where the likelihood of support jumps from 17.44% (non-seniors) to 35.88% (seniors). (We computed the percentages using *Clarify* [32].) These are substantial changes in support due to differences in career status. Clearly, attitudes differ based on career status and associated environmental influences with the implication being that while still under the auspices of the university, student-athletes' expressed opinions follow the perceived NCAA′s and university's stances.

**Table 1:** Predicting Athlete Opinion on Pay for Play, Unionization, and Compliance Worries.

	(1) Pay for Play	(2) Union Support
Senior	0.511^**^ (0.080)	0.575^**^ (0.083)
Gender	−0.078 (0.075)	0.058 (0.077)
African American	0.560^**^ (0.163)	0.375^*^ (0.167)
Income	−0.034 (0.032)	−0.046 (0.033)
PID	0.032 (0.030)	0.003 (0.031)
Ideology	−0.026 (0.033)	−0.110^**^ (0.035)
Football	0.423^**^ (0.146)	0.384^*^ (0.149)
Male Basketball	0.957^*^ (0.439)	0.357 (0.403)
Athletic Scholarship	0.111 (0.068)	0.068 (0.071)
Cut 1	−0.931 (0.195)	−1.39 (0.204)
Cut 2	−0.391 (0.194)	−0.790 (0.202)
Cut 3	−0.185 (0.194)	−0.583 (0.202)
Cut 4	−0.009 (0.194)	0.519 (0.202)
Cut 5	0.426 (0.194)	0.876 (0.203)
Cut 6	0.977 (0.195)	1.38 (0.207)
*N*	964	888
*Pseudo R2*	0.0225	0.0302

**Notes:** Results for Models 1 and 2 are from ordered probit regressions. Standard errors in parentheses; + p<0.10, * p<0.05, ** p<0.01 (Two-Tailed Tests).

Other than career status, we find that African-Americans are significantly more likely to support both unionization and pay for play. This finding coheres with surveys of the general public that also suggested increased support among African-Americans [34]. Commenting on this dynamic, Northwestern Professor Aldon Morris hypothesizes that “White people who come from privilege are more likely to sympathize with the managerial interests like the University, whereas black people better relate to the working-class perspective, in this case the players” [34]. We additionally find that conservative ideology is associated with significantly less support for unionization, while being a member of the football team is associated with heighted support relative to non-football players. Football and men's basketball team members demonstrate significant increases in support for pay for play.

Our survey included two additional items that offer insight into the dynamics of opinions change. Specifically, we asked respondents whether they had heard of unionization efforts and about the importance of their attitudes. We find that only 13.13% (937) of all respondents had heard of the unionization efforts; however, the results also reveal a significant difference between non-seniors (10.31; 708) and seniors (21.97; 223) (*z* = 4.50; *p*<.01). In line with some theories of social networks, this suggests new networks can involve the transmission of information [9]. That said, the low overall percentages of respondents who knew of unionization efforts highlights how quickly issues can change in the NCAA; as mentioned, our survey occurred prior to the well-publicized unionization effort; we suspect virtually all student-athletes in 2014 would know of these efforts. It is unclear how this has altered the opinion dynamics of each group.

On attitude importance or strength (i.e., “how important are these issues to you,” on a 7-point scale from extremely unimportant to extremely important), we find an overall mean of 4.55 (std. dev.  = 1.68; n = 1063), and a significant difference between non-seniors (4.44; 1.66; 812) and seniors (4.90; 1.70; 242) (*t_1052_* = 3.76; *p*<.01). At first glance, it may seem surprising that seniors view issues that no longer directly affect their lives as more important. The finding is consistent, however, with the idea that student-athletes, during their careers, may feel ambivalence due to an acute social context made up of many individuals opposed to pay for play and unionization mixed with a broader information (media) environment that often supports such measures: – “[a]mbivalence is negatively associate with attitude strength” [10], page 780. Post career, however, student-athletes are more removed from the immediate athletic context and ambivalence declines leading to stronger opinions. (For both knowledge of the union and attitude importance, we find statistical significance are robust to the inclusion of control variables in multivariate analyses; see the Supporting Information, Table S1 in S1 File.)

These results suggest that a shift in social environment, as predicted by the spiral of silence theory, causes individuals to engage in post-career reflection and recognize the importance of rules regulating their playing careers. As a former Division I college football player states, “The more removed from playing you are, the more you look back and shake your head at what you gave the school and what the school gave you” [35]). That said, our results do *not* suggest that the NCAA, athletic conferences, universities, or athletic staffs have militant opinions or that they are taking actions that are intentionally meant to influence the attitudes of student-athletes. Rather, our results suggest that current student-athletes' expressed opinions seemed to be shaped by the university environment, even if the relevant actors have no intent to affect student-athletes' opinions.

Another possible explanation for differential responses based on the point in one's career is a threat of disclosure bias in survey response. In this case, even if a student-athlete holds a particular opinion (for example, support for pay for play and unionization), he or she may not openly express that opinion when asked due to a concern it will become public information [36], [37], [38]. This is unlikely, however, to be behind our results for four reasons [37], pages 255–288. First, the questions themselves do not ask for particularly private information (such as questions, e.g., about drug use, sexual behavior). Second, the survey was self-administered with assurance of anonymity and thus respondents likely did not fear that their individual answers would be revealed to others (e.g., their coaches). Third, we asked respondents about their fear of non-compliance with the NCAA and there was not a significant difference in scores between seniors and non-seniors. Finally, the change in knowledge and attitude importance suggests a substantive shift rather than item misreporting on the pay for play and unionization questions.

Our results say *nothing* about what may be ideal for student-athletes, universities, or the NCAA. Indeed, most indications are the schools often go to extraordinary efforts to encourage student-athletes to think for themselves. For example, in the aftermath of Northwestern's Kain Colter's efforts to unionize, Northwestern University and athletic department officials clearly supported the individual players' rights to support unionization, even though the institution itself opposed unionization [20]; http://www.northwestern.edu/newscenter/stories/2014/04/football-player-unionizationnlrb-information.html]. The school released a statement that explained, “We deeply appreciate each and every one of the young men who came out today and allowed their voices to be heard. They represent what we are all about at Northwestern: intelligent, thoughtful, involved students and leaders who make up their own minds on important matters…. This discussion has put us in the national spotlight. Northwestern strongly believes in the issues that have been raised, and the University has been a leader in several of these areas, including awarding four-year scholarships and providing extended medical benefits” (http://www.northwestern.edu/newscenter/stories/2014/04/statement-by-jim-phillips,-vice-president-for-athletics-and-recreation.html). Furthermore, Pat Fitzgerald, Colter's former coach, while opposing unionization, applauded the courage of his former player [13], [15], [16].

All of this highlights the need for future work. First, there is an obvious need for an updated study of student-athletes' opinions that accounts for recent unionization efforts and reforms such as full cost of attendance scholarships. Additional research is also needed to update and expand our findings to other conferences and universities, including smaller schools and schools that have explicitly cut sports due to revenue burdens or Title IX compliance. Second, research is needed to pin down mediating processes [39]; this would entail exploring the nature of perceived networks (e.g., who exactly makes up the changing networks), the directional opinions of the network, and mechanism through which the networks exert influence (e.g., how does the sociological changes generate psychological consequences?). For example, in some cases, networks shape opinions via information transmission (e.g., learning about unions) whereas in other instances, conformity is at work (e.g., attitudes change in an effort to resemble others due to social pressure) [9], pages 7–13, [10], pages 780–781. Or are other psychological processes at work?

Another question concerns the accuracy of *perceptions* of social environments: if current student-athletes' opinions stem largely from views held by the NCAA, and their teams, coaches, and universities, are their perceptions accurate? As mentioned, many Universities go to great lengths to re-assure players who may hold distinct views – do student-athletes perceive this? If not, why? Is there variance across universities? Related, should student-athletes be in fact concerned about the social isolation of holding minority opinions as the spiral of silence theory purports?

## Conclusions

What do the evident post-career attitude shifts imply? As explained, it does *not* indicate fault with the NCAA, the Big Ten, or universities – it is entirely probable, if not likely, that each entity is working to ensure what they believe is in the best interests of student-athletes. What the post-career shifts in opinion do likely suggest is that student-athletes' opinions are shaped by their *perceived* social environments. During their playing career, that environment consists largely of individuals from their university and team, and this changes after one's career comes to an end. The findings also suggest that post-career ex-student-athletes tend towards believing that reforms would have improved their college experiences. In some sense, these findings should not be surprising given theories of attitude change and social-interaction make very clear that such interactions can have a dramatic effect on individuals' opinions [9]; what is novel here is the application to a widely debated area.

We believe there are third key take-away points from this study. First, it is important to recognize the ostensible powerful role the *perceived* social context appears to play in structuring student-athlete opinions, and that opinions expressed during one's career may be shaped to reflect those purportedly held by the NCCA, teams, coaches, and athletic department. As mentioned, the context itself has changed since our study in 2012, and in some ways is changing at a remarkably rapid pace. Of particular note is a recent NCAA reform that enables five conferences, consisting of 65 teams, to have increased autonomy in rule-making which likely will lead to guaranteed four year scholarships, lifetime education opportunity, increased medical insurance, full cost of attendance scholarships, as well as a rule-making body that will include current student-athletes (18.8% of the body will be made up of student-athletes). This reform not only could alter current and former student-athlete opinions but also may drive a wedge between the opinions of those in the five conferences and those in different conferences. More study is clearly needed.

Second, uncovering more precise dynamics about opinion changes would provide insight into whether the opinions of post-career student-athletes should be considered as reforms are debated. Third, our results do *not* suggest that pay for play and unionization are optimal solutions for any of the relevant parties. Both come with a host of questions and complications including compliance with Title IX, equality in payments, and equality between universities. The aforementioned recently adopted reforms may be worthwhile alternative reforms aimed at improving the student-athlete experience (and also ensure universities can continue to financially support non-revenue sports): only future work would tell if these changes have affected the opinions and current and former student-athletes.

What *is* clear is that former student-athletes believe their experiences could have been improved (as is evident by the shift in their opinions in the direction of wanting changes): whether these preferences should be considered and/or are even accurate in terms of what would have made for a better experience is unclear. Regardless, future studies need to recognize the role of one's social environment in structuring opinions.

## Supporting Information

S1 File **Supporting Information: Contains Table S1 Model Results for Union Knowledge and Importance.**
(DOCX)
