# Supplementary material for: The Role of Social Context in Shaping Student-Athlete Opinions
Source: PLoS One. 2014 Dec 18;9(12):e115159. doi: 10.1371/journal.pone.0115159 (PMC4270728; doi:10.1371/journal.pone.0115159)
Supplement: S1 File — Supporting Information: Contains Table S1 Model Results for Union Knowledge and Importance. (DOCX) [file pone.0115159.s001.docx]

**Appendix**

**Table S1: Model Results for Union Knowledge and Importance**

|  | (1)  Heard of Union  (probit) | (2)  Importance  (ordered probit) |
| --- | --- | --- |
| Senior | 0.477^**^  (0.122) | 0.277^**^  (0.080) |
| Gender | -0.105  (0.126) | 0.103  (0.075) |
| African-American | .020  (0.283) | 0.112  (0.154) |
| Income | -0.002  (0.055) | -0.078*  (0.032) |
| PID | 0.080  (0.051) | 0.002  (0.030) |
| Ideology | -0.031  (0.056) | -0.003  (0.034) |
| Football | 0.683^**^  (0.211) | -0.144  (0.142) |
| Male Basketball | 1.24^*^  (0.492) | -0.173  (0.388) |
| Athletic Scholarship | 0.039  (0.116) | 0.128^+^  (0.069) |
| Constant | -1.41^**^  (0.325) |  |
| Cut 1 |  | -1.90  (0.209) |
| Cut 2 |  | -1.03  (0.198) |
| Cut 3 |  | -0.632  (0.197) |
| Cut 4 |  | -0.122  (0.197) |
| Cut 5 |  | 0.427  (0.197) |
| Cut 6 |  | 1.12  (0.199) |
| *N* | 821 | 915 |
| *Pseudo R2* | 0.0621 | 0.0086 |

***Notes***: Standard errors in parentheses; + p < 0.10, * p < 0.05, ** p < 0.01 (Two-Tailed Tests)
